# Evaluation of LRP6, SFRP3, and DVL1 Protein Concentrations in Serum of Patients with Gastroenteropancreatic or Bronchopulmonary Neuroendocrine Tumors

**DOI:** 10.3390/cancers17010047

**Published:** 2024-12-27

**Authors:** Roksana Duszkiewicz, Janusz Strzelczyk, Elżbieta Chełmecka, Joanna Katarzyna Strzelczyk

**Affiliations:** 1 Department of Medical and Molecular Biology, Faculty of Medical Sciences in Zabrze, Medical University of Silesia, 19 Jordana St., 41-808 Zabrze, Poland; 2 Department of Endocrinology and Neuroendocrine Tumors, Department of Pathophysiology and Endocrinology, Faculty of Medical Sciences in Zabrze, Medical University of Silesia 35 Ceglana St., 40-514 Katowice, Poland; 3 Department of Medical Statistics, Faculty of Pharmaceutical Sciences in Sosnowiec, Medical University of Silesia, 30 Ostrogórska St., 41-200 Sosnowiec, Poland

**Keywords:** neuroendocrine tumor, NET, low-density lipoprotein receptor-related protein 6, LRP6, secreted frizzled-related protein 3, SFRP3, segment polarity protein dishevelled homolog, DVL1, serum, ELISA, gastroenteropancreatic tumor, GEP-NET, bronchopulmonary tumor, BP-NET

## Abstract

**Introduction:** Neuroendocrine tumors are a diverse group of tumors predominantly found in the gastrointestinal tract or respiratory system. **Methods**: This retrospective study aimed to measure the serum concentrations of LRP6 (low-density lipoprotein receptor-related protein 6), SFRP3 (secreted frizzled-related protein 3), and DVL1 (segment polarity protein dishevelled homolog) using the ELISA method in patients with NETs (N = 80) and a control group (N = 62). We evaluated the results against various demographic, clinicopathological, and biochemical characteristics. **Results**: Our analyses revealed that the concentration of SFRP3 in patients with neuroendocrine tumors was significantly elevated (*p* < 0.001) compared to the control group. Additionally, DVL1 concentrations were significantly higher (*p* < 0.01) in patients with BP-NETs compared to GEP-NETs. Furthermore, DVL1 analysis showed a moderate negative correlation with chromogranin A (*p* < 0.001) and weak negative correlations with serotonin (*p* < 0.05) and 5-HIAA (*p* < 0.05). Significant negative correlations were also observed between DVL1 and age in the control group (*p* < 0.01), and between LRP6 and Ki-67 in the study group. **Conclusions:** These results suggest that changes in the SFRP3 and DVL1 pathways play a key role in NET development. Elevated levels of these proteins highlight their importance in tumor biology, with SFRP3 and DVL1 potentially being crucial in NET molecular mechanisms. Further research is needed to explore their roles and potential in diagnosis and treatment.

## Simple Summary

This retrospective study investigated the serum levels of LRP6, SFRP3, and DVL1 in patients with neuroendocrine tumors (NETs) and a control group using the ELISA method. The results showed significantly elevated SFRP3 levels in NET patients compared to controls and higher DVL1 levels in BP-NETs than GEP-NETs. DVL1 was negatively correlated with chromogranin A, serotonin, 5-HIAA, and age in controls. LRP6 was negatively correlated with Ki-67 in NET patients. These findings suggest that the SFRP3 and DVL1 pathways are important in NET development, highlighting their potential in diagnosis and treatment. Further research is needed to explore these roles.

## 1. Introduction

Neuroendocrine tumors (NETs) originate from neuroendocrine cells and represent a diverse group of solid tumors. They are most frequently found in the gastrointestinal tract and respiratory system, with less common occurrences in the genitourinary system [1,2]. These tumors are challenging to diagnose due to their heterogeneity and often indolent nature. Biochemical diagnostics for NETs include both non-specific and specific markers. Serum chromogranin A is a widely used non-specific marker, while specific markers such as serotonin and 5-hydroxyindole acetic acid (5-HIAA) are essential for detecting certain types of NETs [3]. The selection of these markers depends largely on the type and location of the suspected tumor, which aids in tailoring the diagnostic approach [3,4]. The field of NET diagnostics and treatment is evolving, with ongoing research focusing on identifying new predictive markers [5]. These studies aim to enhance early detection, improve prognostic evaluations, and tailor treatments more effectively. Despite advancements, the complexity and variability of NETs necessitate the continuous exploration of innovative diagnostic and therapeutic strategies to better manage this challenging group of tumors. According to the eighth edition of the AJCC/UICC (American Joint Committee on Cancer/Union for International Cancer Control) recommendations, the ESMO (European Society for Medical Oncology) guidelines, and the 2022 WHO (World Health Organization) classification, NETs are defined as NET G1 (Ki-67 < 3%), NET G2 (Ki-67 3–20%), NET G3 (Ki-67 > 20%, usually between 21–55%) and NEC (neuroendocrine carcinoma, Ki-67 usually > 55%) (i.e., low-differentiated carcinomas with a highly aggressive clinical course) [6,7,8]. Based on the 2021 WHO classification of lung tumors, bronchopulmonary neuroendocrine tumors (BP-NETs) are categorized into four groups: typical carcinoids (neuroendocrine tumors grade 1), atypical carcinoids (neuroendocrine tumors grade 2), large cell neuroendocrine carcinomas, and small cell carcinomas [9].

This study involved evaluating the concentration in serum of low-density lipoprotein receptor-related protein 6 (LRP6), secreted frizzled-related protein 3 (SFRP3), and segment polarity protein dishevelled homolog 1 (DVL1) in patients with NETs.

LRP6, a member of the low-density lipoprotein receptor family, exhibits a distinctive structure that enables its multifaceted roles as both a co-receptor in the Wnt/β-catenin signaling pathway and a receptor for ligand-mediated endocytosis [10,11,12]. Recent studies have highlighted its significance in metabolic regulation, particularly within nutrient-sensing pathways [13,14]. The Wnt signaling pathway, essential for regulating gene expression, cell proliferation, and migration during embryonic development and tumorigenesis, involves the binding of Wnt protein ligands to receptors of the FZD family [15,16,17]. This pathway is categorized into canonical and non-canonical signaling branches [13]. Canonical Wnt signaling is mediated by the FZD and LRP5/6 receptors, while non-canonical signaling involves FZD receptors and other co-receptors, influencing downstream Wnt/planar cell polarity, Wnt/receptor tyrosine kinase, and Wnt/calcium pathways [18,19]. In canonical Wnt signaling, the binding of Wnt proteins (e.g., Wnt1 or Wnt3) to their receptors, including members of the FZD family and LRP5/6, initiates a cascade leading to the phosphorylation of the adaptor protein Dishevelled [10,18]. This phosphorylation promotes DVL’s interaction with Axin, an inhibitor of the Wnt signaling pathway, thereby modulating the pathway’s activity [20,21].

SFRP3 modulates the Wnt signaling pathway through direct interaction with Wnt proteins. These proteins are crucial in regulating cell growth and differentiation in specific cell types, including the maturation of chondrocytes and the development of long bones [22]. LRP6 acts as a co-receptor and is essential for the activation of the canonical Wnt signaling pathway. Together with the Wnt and FZD proteins, LRP6 forms a complex that facilitates signal transduction. These proteins exhibit both paracrine and endocrine effects [15,23,24].

The DVL1 proteins could play a crucial role in the signal transduction pathways activated by various Wnt proteins [25]. They are involved in both canonical and non-canonical Wnt signaling pathways by binding to the cytoplasmic C-terminus of FZD family proteins and transmitting signals to downstream effectors. Additionally, DVL1 promotes the internalization and degradation of FZD proteins following Wnt signaling [18,26,27,28].

These three proteins are integral components of the canonical Wnt signaling pathway, which is crucial in regulating cellular metabolism [10,18]. Thus, analyzing the concentrations of these proteins in the blood serum of patients with NETs is well justified. According to available data, this study is among the few that has evaluated LRP6, SFRP3, and DVL1 in NET patients. However, no previous studies have used ELISA tests for this analysis.

The aim of this study was to evaluate and compare the serum concentrations of LRP6, SFRP3, and DVL1 between patients with neuroendocrine tumors (NETs) and healthy individuals, and to correlate these findings with selected demographic, clinicopathological, and biochemical characteristics. Additionally, we analyzed and compared the concentrations of these proteins in patients with gastroenteropancreatic neuroendocrine tumors (GEP-NETs) or bronchopulmonary neuroendocrine tumors (BP-NETs), correlating these results with their respective characteristics.

## 2. Materials and Methods

### 2.1. Patients

**Figure 1:**
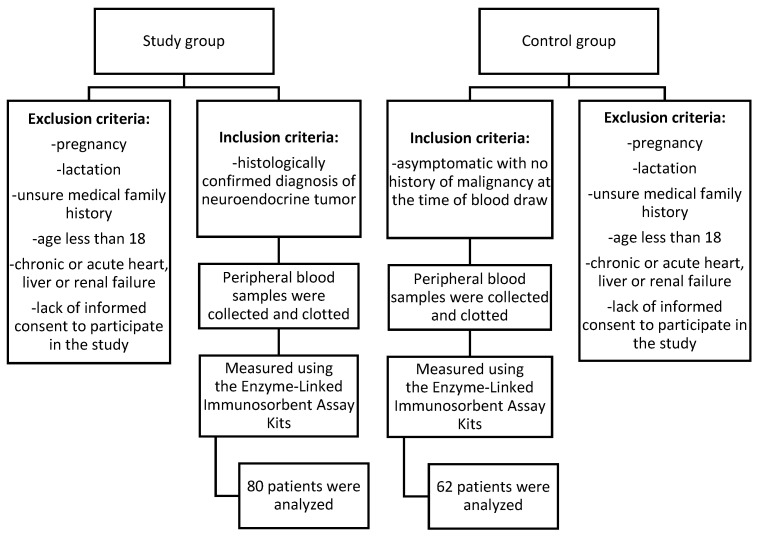
Flow chart of the patients included in the study.

Patients for this study were recruited from the Department of Endocrinology and Neuroendocrine Tumors, Department of Pathophysiology and Endocrinology at the Medical University of Silesia in Katowice, Poland. The main inclusion criteria for both the study and control groups were being aged 18 or older and providing signed consent to participate, with the study group specifically including individuals with a histologically confirmed diagnosis of a neuroendocrine tumor and not undergoing chemotherapy, and a control group including healthy volunteers with no history of malignancy at the time of blood draw. Details are presented in Table 1 and Figure 1. All patients from the study group underwent core needle biopsy or histopathological examination after surgery. In each case, the diagnosis was verified during hospitalization to ensure accuracy. This aligned with the recommendation for biopsy confirmation as highlighted in previous studies [29,30]. Patients were newly diagnosed and had not undergone any prior treatment. The only procedures performed were core needle biopsies or surgical interventions for diagnostic purposes. After reviewing medical records, the information on patients’ neuroendocrine markers, including chromogranin A, serotonin, and 5-HIAA, was gathered.

Histological confirmation of the NETs was performed by experienced pathologists who confirmed the tumors according to the WHO 2019 [31] classification for GEP-NETs and the WHO 2015 [9] classification for BP-NETs, with tissue specimens analyzed using hematoxylin and eosin (H&E) staining and immunohistochemistry.

The extent of the disease was assessed using anatomical imaging methods, including computed tomography (CT) and/or magnetic resonance imaging (MRI), along with functional imaging such as [^68^Ga]Ga-DOTATATE PET/CT for well-differentiated NETs and [^18^F]FDG PET/CT for G2/G3 NETs. All imaging studies were evaluated by specialists in radiology or nuclear medicine. Gastroscopy and colonoscopy were utilized for assessing gastroenteropancreatic (GNETs) and rectal neuroendocrine tumors (RNETs).

### 2.2. Funding Statement and Ethics Approval and Consent to Participate

This study was conducted in accordance with the Declaration of Helsinki and approved by the Bioethics Committee of the Medical University of Silesia (20 September 2022 No. PCN/CBN/0052/KB1/24/II/22).

### 2.3. Study Design

Peripheral blood samples were collected into S-Monovette tubes with a clotting activator (Sarstedt, Germany). To obtain serum, the blood was centrifuged for 15 min at 3500 rpm, and the serum was subsequently stored at −80 °C for further analysis. The serum concentrations of LRP6, SFRP3, and DVL1 were assessed using ELISA, following the procedures described by the manufacturers. The analytical procedure adhered to the manufacturer’s guidelines as outlined in the technological manuals provided with the kits.

All ELISA tests were performed at the Department of Medical and Molecular Biology, Faculty of Medical Sciences in Zabrze, Medical University of Silesia in Katowice, Poland. To determine the concentrations of the tested samples, a standard curve was prepared using the standards provided with the kits. All standards and serum samples were run in duplicate, with serum samples diluted as specified by the instructions (2× for LRP6, undiluted for SFRP3, and 2× for DVL1). The ELISA plates were read using a Bio-Tek µQuant Universal Microplate Spectrophotometer (Bio-Tek Instruments, Inc., Winooski, VT, USA) at a primary wavelength of 450 nm. Data analysis was conducted using KCJunior Software (Bio-Tek Instruments, Inc., Winooski, VT, USA), https://www.agilent.com/ URL (accessed on 30 March 2023) and absorbance values were converted to concentrations in ng/mL for all three proteins measured.

The assay kits used were from Wuhan Fine Biotech Co., Ltd., Wuhan, China, with product numbers EH1124 for LRP6, EH8604 for SFRP3, and EH1769 for DVL1. The minimum detectable doses were 0.188 ng/mL for LRP6, 0.094 ng/mL for SFRP3, and 0.094 ng/mL for DVL1.

### 2.4. Statistical Analysis

Qualitative data are expressed as percentages. For quantitative data, the Shapiro–Wilk test was used to assess the normality of the data distribution. Normally distributed data are reported as mean ± standard deviation (M ± SD). For non-normally distributed data, medians with interquartile ranges (Me (Q_1_–Q_3_)) are provided. Comparisons between variables were made using the Student’s *t*-test, the Mann–Whitney U test, or the non-parametric Kruskal–Wallis test. The Spearman correlation coefficient was utilized to evaluate the relationships between variables. To compare the levels of the analyzed parameters between the BP-NET and GEP-NET groups, the receiver operating characteristic (ROC) curve analysis was performed. In these analyses, BP-NET was considered as positive and GEP-NET was considered as negative. In the ROC curve analysis, the cut-off point was determined by analyzing the Youden index (J) values. This was calculated according to the following formula: J = sensitivity + specificity − 1. The Youden index can take values from 0 to 1. The most optimal point was characterized by the highest J value and is located closest to the upper, left part of the graph. A significance threshold of 0.05 was set for all tests, which were conducted as two-sided. Statistical analysis was carried out using Statistica software (TIBCO Software Inc. (2017), Statistica (data analysis software system), version 13. https://www.statsoft.pl, URL (accessed on 17 April 2024).

## 3. Results

### Characteristics of the Study Groups

This study included 142 participants in total. Among them, 56% (N = 80) were in the study group, consisting of patients with NETs. The remaining 44% of participants (N = 62) formed the control group and were healthy individuals with no history of malignancy at the time of blood collection. In the study group, 20% of patients were diagnosed with BP-NET, whereas 80% were diagnosed with GEP-NET. Additional details can be found in Table 1.

There was no significant difference in age (*p* = 0.083) or BMI (*p* = 0.385) between the study and control groups. Additionally, the sex distribution did not differ significantly between the groups (*p* = 0.343). No significant associations were found for hypertension (*p* = 0.205) or smoking status (*p* = 0.911). However, a significant correlation was observed for diabetes (*p* < 0.05). Details are presented in Table 2.

**Figure 2:**
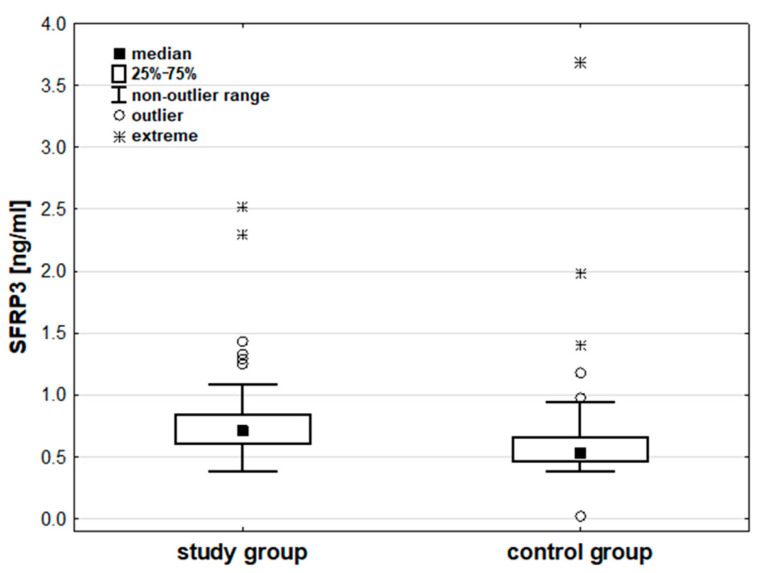
The concentration of SFRP3 [ng/mL] in the study and control groups.

Analysis of the measured parameter concentrations indicated no significant differences in LRP6 (*p* = 0.486) and DVL1 (*p* = 0.058) concentrations between the groups. SFRP3 concentrations were significantly higher in the study group than in the control group (*p* < 0.001). Additional details can be found in Table 3 and Figure 2. Details are also presented in Table S1 in the Supplementary Materials.

No significant differences were found between the protein concentrations and age, BMI, Ki-67, or clinical stage for LRP6, SFRP3, and DVL1. However, a significant negative correlation was observed between DVL1 and age in the control group (*p* < 0.01), as well as between LRP6 and Ki-67 in the study group. Details are shown in Table 4. Additionally, no significant differences were found between the protein concentrations and other parameters such as sex, histological grade, metastasis, liver metastasis, lymph node metastasis, or bone metastasis.

**Figure 3:**
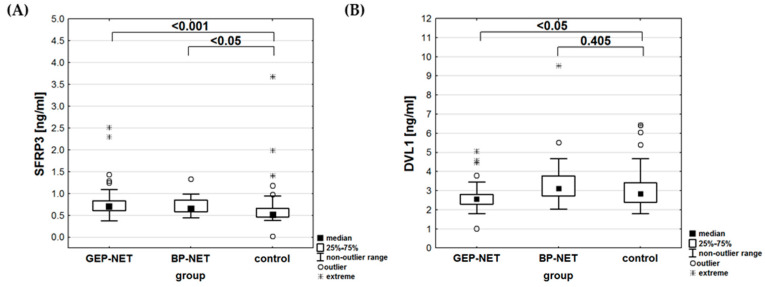
The concentration of SFRP3 [ng/mL] (**A**) and DVL1 [ng/mL] (**B**) in the study and control groups, indicating statistical significance between the location of the primary tumor and the control group.

**Figure 4:**
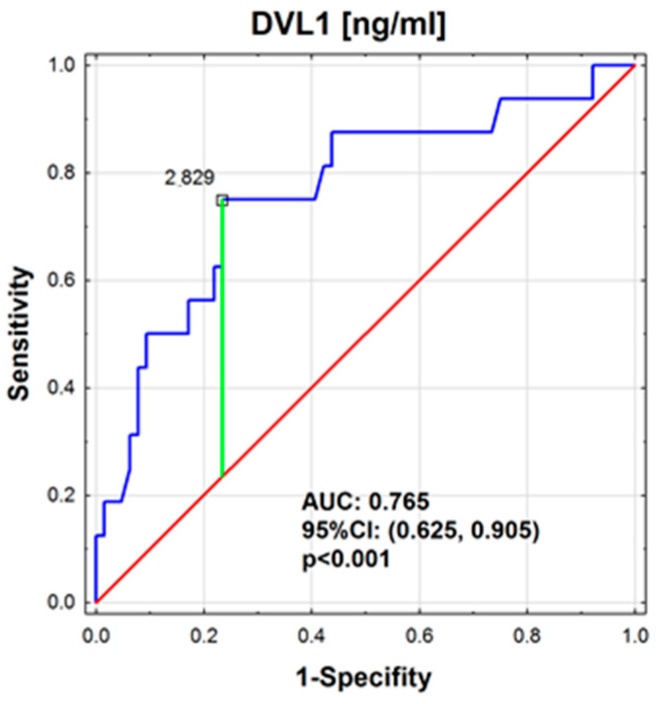
ROC curve for determining the DVL1 concentration [ng/mL] in the location of the primary tumor (GEP-NET and BP-NET).

The analysis showed no significant differences in LRP6 (*p* = 0.373) or SFRP3 (*p* = 0.601) concentrations based on the location of the primary tumor. However, DVL1 concentrations varied significantly (*p* < 0.01). The highest concentration of SFRP3 was observed in the BP-NET and GEP-NET groups, with statistically significant differences compared to the control group (Table 5, Figure 3A). In contrast, the concentration of DVL1 reached its highest levels in the BP-NET group, while the GEP-NET group and the control did not show any statistical differences (Figure 3B). An analysis of the area under the ROC curve was performed for the patients. For DVL1, the area under the ROC curve was significantly greater than 0.5 (*p* < 0.001), with a cutoff point qualifying patients into the BP-NET group at 2.83 ng/mL (Figure 4). For SFRP3 and LRP6, the areas under the ROC curves did not statistically differ from the value of 0.5 (*p* = 0.609 and *p* = 0.396, respectively).

When examining specific primary tumor sites (lung, pancreas, small intestine, duodenum, stomach, rectum), there were no significant differences in LRP6 (*p* = 0.333) and SFRP3 (*p* = 0.154) concentrations, but DVL1 results showed significant variation (*p* < 0.01). Further comparisons revealed significant differences between the pancreas and lung (*p* < 0.05) and between the small intestine and lung (*p* < 0.05).

There was no significant correlation between LRP6 or SFRP3 concentrations and the concentrations of chromogranin A, serotonin, or 5-HIAA. Notably, DVL1 showed a negative correlation with chromogranin A (*p* < 0.001) and weak negative correlations with serotonin (*p* < 0.05) and 5-HIAA (*p* < 0.05). Further details are provided in Table 6. Additionally, protein concentrations did not correlate with glucose, TCH, or TG concentrations.

In comparing the concentrations of LRP6, SFRP3, and DVL1 between smokers (study group N = 9) and non-smokers (control group N = 12), no statistically significant differences were found (LRP6: z = 0.618, *p* = 0.537; SFRP3: z = 1.866, *p* = 0.187; DVL1: z = 1.325, *p* = 0.185). Moreover, analysis of the relationships between these protein concentrations revealed no significant correlations between SFRP3 and LRP6 (ρ = 0.146, *p* = 0.203) or between SFRP3 and DVL1 (ρ = 0.020, *p* = 0.857). However, there was a weak positive correlation between LRP6 and DVL1 (ρ = 0.284, *p* < 0.05).

## 4. Discussion

The rising incidence of NETs poses a significant medical challenge, as advanced stages remain incurable and often fatal [32,33,34]. Despite extensive research, effective treatments are limited. NETs typically originate in the gastroenteropancreatic system and lungs [35]. Treatment for inoperable or advanced NETs depends on factors such as tumor site, grade, the extent of metastasis, hormonal activity, and genetic characteristics [36,37]. Although there have been advancements in understanding NET genetics and the introduction of targeted therapies, new systemic treatments for advanced NETs are still critically needed [38,39].

A recent in vitro study explored targeting the Wnt/β-catenin signaling pathway as a novel therapeutic approach for NETs, highlighting its crucial role in regulating tumor cell growth and invasion [11,18,40,41]. The menin protein, encoded by the MEN1 gene, negatively regulates β-catenin, underscoring the pathway’s importance in NET biology [42]. The Wnt signaling pathway, essential for gene expression, cell proliferation, and migration during development and cancer, involves 19 Wnt ligands and 10 FZD receptors [11,43,44]. This pathway is divided into canonical and non-canonical categories: canonical signaling involves Wnt proteins binding to FZD receptors and LRP5/6, leading to β-catenin accumulation and gene expression [11,45], while non-canonical signaling, not involving β-catenin, activates pathways like Wnt/planar cell polarity, Wnt/receptor tyrosine kinase, and Wnt/calcium, influencing various cellular processes through ligands such as Wnt5a/b and Wnt11 [11,46,47,48].

Our results indicate no statistically significant differences in LRP6 concentrations between the study and control groups. Currently, no studies have investigated the concentration of LRP6 in patients with NETs or in animal models. Existing research on LRP6 is limited to studies involving epithelial cancers, such as colorectal, liver, breast, and pancreatic cancers, often using immunohistochemical tests or bioinformatic analysis [49,50,51,52,53]. The lack of statistically significant differences in LRP6 concentrations, combined with limited research, underscores the need for further studies to understand its role in neuroendocrine tumors. This knowledge gap presents an opportunity for future investigations to provide deeper insights into its potential significance.

Our study also explored potential relationships between protein concentrations and various demographic and clinical factors. In the study group of NET patients, we observed a significant negative correlation between LRP6 concentration and the Ki-67 index (*p* < 0.05). The Ki-67 index, a marker of cell proliferation, suggests that lower LRP6 concentrations in tumors with higher Ki-67 indices may indicate that reduced LRP6 concentration is associated with more aggressive, rapidly proliferating tumors. While detailed studies on LRP6 in NETs are lacking, findings from other cancers suggest that LRP6 overexpression may contribute to tumor aggressiveness [52,54]. For example, in pancreatic neuroendocrine tumors (PNETs), dysregulated pathways involving LRP6 are associated with tumor growth and progression [55]. This relationship could be crucial for understanding LRP6′s role in tumor growth and progression, potentially making it a target for therapeutic intervention in highly proliferative NETs [56,57,58,59,60].

The results of our analyses for SFRP3 revealed significant differences between the study and control groups (*p* < 0.001). In-depth comparisons indicated that both the GEP-NET and BP-NET groups differed from the control group. The BP-NET and GEP-NET groups showed the highest SFRP3 concentrations with statistically significant differences compared to the control group. Comparing the BP-NET and GEP-NET groups with each other did not yield any significant conclusions. This result suggests a potential role for SFRP3 in the NET development or progression. There are no studies on SFRP3 concentration in NET patients or animal models using immunoenzymatic methods. SFRP3 functions as an inhibitor of the Wnt/β-catenin pathway, which is crucial for cell growth, differentiation, and apoptosis [61]. In hepatocellular carcinoma (HCC), SFRP3 overexpression has been shown to inhibit tumor growth by inactivating the Wnt/β-catenin signaling pathway [62]. This led to reduced cell proliferation and increased differentiation [63]. Additionally, in rhabdomyosarcoma, particularly the alveolar subtype, the suppression of SFRP3 has been found to inhibit cell growth and induce apoptosis [63,64]. This highlights SFRP3′s role in supporting tumorigenesis by modulating the Wnt signaling pathway. Targeting SFRP3 could therefore be a potential therapeutic strategy in cancers where Wnt signaling is dysregulated [62,63,64]. While specific studies directly linking SFRP3 and neuroendocrine tumor proliferation are limited, mechanisms observed in other cancer types suggest that SFRP3 likely influences neuroendocrine tumor growth through similar pathways [61]. Understanding the role of SFRP3 in these processes could provide insights into new therapeutic approaches for managing highly proliferative neuroendocrine tumors.

DVL1 concentrations varied significantly (*p* < 0.01), with higher concentrations observed in BP-NETs compared to GEP-NETs. For DVL1, only the GEP-NET group had lower concentrations compared to healthy individuals. In this case, the ROC curve analysis enabled the determination of a cutoff point between the GEP-NET and BP-NET groups, demonstrating differences in the concentrations between these groups. DVL1 is another critical component of the Wnt signaling pathway in BP-NETs, and its elevated concentration in serum could indicate abnormal activation of this pathway. Such dysregulation may contribute to tumor progression and suggests that the Wnt pathway is functioning in a disrupted manner, potentially influencing cell proliferation and metastasis in NETs. When examining specific primary tumor sites, including the lung, pancreas, small intestine, duodenum, stomach, and rectum, our analysis revealed significant variation in DVL1 concentrations (*p* < 0.01). This indicated that the DVL1 concentration is not uniform across the different types of neuroendocrine tumors and may reflect the unique biological characteristics of tumors originating in different organs [65]. Further comparisons between these sites provided more detailed insights. For instance, DVL1 concentrations were significantly different between pancreatic and lung NETs (*p* < 0.05), suggesting that the molecular mechanisms involving DVL1 in pancreatic NETs might differ from those in lung NETs. Similarly, significant differences were observed between small intestine NETs and lung NETs (*p* < 0.05), pointing to distinct regulatory pathways that influence DVL1 concentration. There are currently no studies on the concentration of DVL1 in patients with neuroendocrine tumors, nor have any been conducted using animal models to explore this connection. However, DVL1 has been studied in other types of cancers, suggesting its potential role in cancer biology and signaling pathways, which might be relevant for future investigations in NETs [66,67,68,69,70]. The variations in DVL1 concentrations across different primary tumor sites could have important implications for understanding the pathophysiology of NETs. For example, the elevated DVL1 concentrations in pancreatic NETs compared to lung NETs might indicate a higher degree of Wnt signaling pathway activation in pancreatic tumors. This could contribute to differences in tumor behavior, such as growth rate, metastatic potential, and response to therapy [36,71,72]. Moreover, these findings highlight the potential of DVL1 as a protein for distinguishing between NETs of different origins. By identifying the specific patterns of DVL1 concentration, clinicians could improve the accuracy of NET diagnosis and potentially tailor treatments based on the tumor’s primary site [26,36,73].

The significant variation in DVL1 concentrations across different primary tumor sites underscores the complexity of neuroendocrine tumors and the need for a nuanced approach to their study and treatment [74]. The analyses of DVL1 revealed a moderate negative correlation with chromogranin A (CgA), a well-known marker for NETs (*p* < 0.001). This suggests that as DVL1 concentrations increase, CgA concentrations tend to decrease, highlighting a complex interplay between these molecules. Additionally, weak negative correlations were also found between DVL1 and serotonin (*p* < 0.05) and DVL1 and 5-HIAA (*p* < 0.05), indicating potential links between Wnt signaling and neuroendocrine function. While no significant associations with age, Ki-67, or clinical stage were found overall, a significant negative correlation between DVL1 and age (*p* < 0.01) was observed in the control group, indicating a possible age-related decline in DVL1 concentration. These findings enhance our understanding of NET molecular diversity and suggest new therapeutic strategies.

These results provide a solid foundation for future research. Further studies should aim to elucidate the precise mechanisms by which SFRP3 and DVL1 contribute to neuroendocrine tumorigenesis. Additionally, investigating these pathways could reveal novel therapeutic targets, ultimately improving patient outcomes. The data presented in this study represent an important step toward a deeper understanding of the molecular underpinnings of neuroendocrine tumors and pave the way for more effective clinical interventions.

## 5. Advantages of the Study

According to the available data, this is one of the few studies evaluating serum LRP6, SFRP3, and DVL1 in NET patients.

## 6. Limitations of the Study

This study has certain limitations. Notably, no information on patient survival was available, so these data could not be included in the analyses. However, these issues will be addressed in future research. Considering the aim of this study and the fact that the correlations pertain to only one group, and since the purpose of this work is not to predict outcomes between the study and control groups, the authors opted not to perform logistic regression. Instead, Spearman’s rank correlation was used to assess the relationships between continuous variables. This study aimed to verify the concentrations of LRP6, SFRP3, and DVL1 and to highlight differences in these parameters among patients from the BP-NET and GEP-NET groups as well as a control group of healthy individuals. Due to the limited number of patients in each group, this study did not aim to validate the analyzed parameters.

## 7. Conclusions

This study highlights the critical role of the Wnt/β-catenin signaling pathway in the development and progression of neuroendocrine tumors (NETs). In conclusion, this study underscores the significant involvement of SFRP3 and DVL1 in the development and progression of neuroendocrine tumors. The elevated serum concentrations of SFRP3 in NET patients, along with the higher DVL1 levels in BP-NETs compared to GEP-NETs, suggest that these proteins may play crucial roles in tumor biology. The observed correlations with clinical markers such as chromogranin A, serotonin, and Ki-67 further emphasize their potential relevance. Moreover, the lack of extensive research on these proteins in NETs underscores the need for further studies to elucidate their precise roles. Understanding these mechanisms could lead to novel therapeutic targets and improve patient outcomes. Overall, this research provides a valuable foundation for advancing the molecular understanding of NETs.

## Figures and Tables

**Table 1:** Characteristics of the study group.

Category	Number of Patients (N = 80)	Percentage (%)
**Study Group Details**		
BP-NET	16	20
GEP-NET	64	80
**GEP-NET Tumor Locations**		
Pancreas	24	30
Small Intestine	21	26
Stomach	8	10
Rectum	7	9
Duodenum	4	5
**Histological Grade (GEP-NET)**		
NET G1	40	50
NET G2	21	26
NET G3	3	4
**BP-NET Types**		
Typical Carcinoid	10	11
Atypical Carcinoid	6	8
**Metastasis**		
Total with Metastasis	35	44
Liver Metastasis	20	25
Lymph Node Metastasis	27	34
Bone Metastasis	7	9
**Clinical Stage**		
Stage I	36	45
Stage II	8	10
Stage III	11	14
Stage IV	25	31
**Ki-67 Index**		
<3%	45	56
3–20%	31	39
>21–55%	4	5

**Table 2:** Comparison of the demographic characteristics between the study and the control groups.

		Control Group	Study Group	*p*
Age [years]		52.7 ± 11.5 *	56.2 ± 12.5 *	0.083
BMI [kg/m^2^]		26.3 ± 4.1 *	26.2 ± 5.3 *	0.385
Sex	Male	23 (37%) ** 39 (63%) **	36 (45%) ** 44 (55%) **	0.343
	Female			
Hypertension		17 (27%) **	30 (38%) **	0.205
Smoking status		9 (14%) **	12 (15%) **	0.911
Diabetes		2 (3%) **	13 (16%) **	<0.05

Legend: * M ± SD—mean ± standard deviation, ** n (%), *p*—statistical significance.

**Table 3:** Comparison of the LRP6, SFRP3, and DVL1 concentrations between the study and control groups.

Parameters	Control Group Me (Q_1_–Q_3_)	Study Group Me (Q_1_–Q_3_)	z	*p*
LRP6 [ng/mL]	5.7 (4.5–7.6)	5.5 (4.5–6.6)	0.70	0.486
SFRP3 [ng/mL]	0.53 (0.46–0.66)	0.70 (0.60–0.84)	5.16	<0.001
DVL1 [ng/mL]	2.8 (2.4–3.4)	2.6 (2.3–3.0)	1.89	0.058

Legend: Me (Q_1_–Q_3_)—median (lower–upper quartile); z—Mann–Whitney U test statistic value, *p*—statistical significance.

**Table 4:** Correlation values between age, BMI, Ki-67, clinical stage and the measured parameters for the study and control groups.

Parameters		Control Group		Study Group	
		ρ	*p*	ρ	*p*
**Age**	**LRP6 [ng/mL]**	−0.092	0.479	−0.115	0.315
	**SFRP3 [ng/mL]**	0.237	0.064	0.032	0.780
	**DVL1 [ng/mL]**	0.343	<0.01	−0.062	0.582
**BMI**	**LRP6 [ng/mL]**	−0.038	0.768	0.192	0.092
	**SFRP3 [ng/mL]**	0.158	0.221	0.015	0.897
	**DVL1 [ng/mL]**	0.170	0.187	0.115	0.309
**Ki-67**	**LRP6 [ng/mL]**	-	-	0.380	<0.01
	**SFRP3 [ng/mL]**	-	-	0.118	0.387
	**DVL1 [ng/mL]**	-	-	0.112	0.413
**Clinical stage**	**LRP6 [ng/mL]**	-	-	−0.086	0.451
	**SFRP3 [ng/mL]**	-	-	−0.037	0.741
	**DVL1 [ng/mL]**	-	-	−0.103	0.362

Legend: ρ—Spearman’s correlation coefficient, *p*—statistical significance.

**Table 5:** Comparison of the LRP6, SFRP3, and DVL1 concentrations for BP-NET, GEP-NET, and control groups.

Parameters	BP-NET Me (Q_1_–Q_3_)	GEP-NET Me (Q_1_–Q_3_)	Control Me (Q_1_–Q_3_)	*p*
LRP6 [ng/mL]	5.9 (4.4–7.5)	5.4 (4.7–6.4)	5.4 (4.7–6.4)	0.544
SFRP3 [ng/mL]	0.7 (0.6–0.8)	0.7 (0.6–0.8)	0.5 (0.5–0.8)	<0.001
DVL1 [ng/mL]	3.1 (2.7–3.8)	2.6 (2.3–2.8)	2.8 (2.4–3.4)	<0.01

Legend: Me (Q_1_–Q_3_)—median (lower-upper quartile); *p*—statistical significance.

**Table 6:** Correlation between parameters related to known NET markers.

Parameters		Study Group	
		ρ	*p*
**Chromogranin A [µg/L]**	**LRP6**	−0.144	0.208
	**SFRP3**	0.034	0.763
	**DVL1**	−0.384	<0.001
**Serotonin [ng/mL]**	**LRP6**	−0.078	0.507
	**SFRP3**	0.154	0.181
	**DVL1**	−0.262	<0.05
**5-hydroxyindole acetic acid (5-HIAA) [mg/24 h]**	**LRP6**	−0.097	0.399
	**SFRP3**	−0.020	0.860
	**DVL1**	−0.282	<0.05

Legend: ρ—Spearman’s correlation coefficient, *p*—statistical significance.

## Data Availability

The data used to support the findings of this research are available upon request from corresponding author, d200793@365.sum.edu.pl.

## Supplementary Materials

The following supporting information can be downloaded at: https://www.mdpi.com/article/10.3390/cancers17010047/s1, Table S1: Description statistics of LRP6, SFRP3, and DVL1.
