# Supplementary material for: Evaluation of LRP6, SFRP3, and DVL1 Protein Concentrations in Serum of Patients with Gastroenteropancreatic or Bronchopulmonary Neuroendocrine Tumors
Source: Cancers (Basel). 2024 Dec 27;17(1):47. doi: 10.3390/cancers17010047 (PMC11718808; doi:10.3390/cancers17010047)
Supplement: Supplementary file 1 [file cancers-17-00047-s001.zip › cancers-3234498-supplementary.pdf]

cancers MDPI

Supplementary materials

Table S1. Description statistics of LRP6, SFRP3 and DVL1

Parameters	Group	M	SD	x_{min}	x_{max}
**LRP6 [ng/ml]**	**Overall**	6.6	5.1	1.9	47.5
	**Study group**	6.4	3.7	3.2	26.9
	**Control group**	6.9	6.4	1.9	47.5
**SFRP3 [ng/ml]**	**Overall**	0.72	0.41	0.03	3.68
	**Study group**	0.78	0.33	0.38	2.52
	**Control group**	0.64	0.47	0.02	3.68
**DVL1 [ng/ml]**	**Overall**	3.0	1.0	1.0	9.5
	**Study group**	2.9	1.0	1.0	9.5
	**Control group**	3.1	1.0	1.8	6.4

Legend: M – mean, SD – standard deviation, x_{min} – minimum, x_{max} – maximum
